# Structure and catalytic mechanism of monodehydroascorbate reductase, MDHAR, from *Oryza sativa* L. *japonica*

**DOI:** 10.1038/srep33903

**Published:** 2016-09-22

**Authors:** Ae Kyung Park, Il-Sup Kim, Hackwon Do, Byung Wook Jeon, Chang Woo Lee, Soo Jung Roh, Seung Chul Shin, Hyun Park, Young-Saeng Kim, Yul-Ho Kim, Ho-Sung Yoon, Jun Hyuck Lee, Han-Woo Kim

**Affiliations:** 1 Division of Polar Life Sciences, Korea Polar Research Institute, Incheon 21990, Republic of Korea; 2 School of Life Sciences, BK21 Plus KNU Creative BioResearch Group, Kyungpook National University, Daegu 41566, Republic of Korea; 3 Department of Polar Sciences, University of Science and Technology, Incheon 21990, Republic of Korea; 4 Research Institute for Ulleung-do & Dok-do, Kyungpook National University, Daegu 41566, Republic of Korea; 5 Highland Agriculture Research Institute, National Institute of Crop Science, Rural Development Administration, Pyeongchang 25342, Republic of Korea

## Abstract

Ascorbic acid (AsA) maintains redox homeostasis by scavenging reactive oxygen species from prokaryotes to eukaryotes, especially plants. The enzyme monodehydroascorbate reductase (MDHAR) regenerates AsA by catalysing the reduction of monodehydroascorbate, using NADH or NADPH as an electron donor. The detailed recycling mechanism of MDHAR remains unclear due to lack of structural information. Here, we present the crystal structures of MDHAR in the presence of cofactors, nicotinamide adenine dinucleotide (NAD^+^) and nicotinamide adenine dinucleotide phosphate (NADP^+^), and complexed with AsA as well as its analogue, isoascorbic acid (ISD). The overall structure of MDHAR is similar to other iron-sulphur protein reductases, except for a unique long loop of 63–80 residues, which seems to be essential in forming the active site pocket. From the structural analysis and structure-guided point mutations, we found that the Arg320 residue plays a major substrate binding role, and the Tyr349 residue mediates electron transfer from NAD(P)H to bound substrate via FAD. The enzymatic activity of MDHAR favours NADH as an electron donor over NADPH. Our results show, for the first time, structural insights into this preference. The MDHAR-ISD complex structure revealed an alternative binding conformation of ISD, compared with the MDHAR-AsA complex. This implies a broad substrate (antioxidant) specificity and resulting greater protective ability of MDHAR.

Reactive oxygen species (ROS) are produced as a normal by-product of aerobic metabolism in living organisms. In addition, various environmental stress conditions including the light/dark cycle, nutrient depletion/excess, high salinity, drought, flooding, extreme temperatures, heavy metals, and UV irradiation are also known to accelerate the accumulation of ROS in plant cells, ultimately leading to cell death^1^,^2^. Therefore, ROS levels must be tightly regulated in order to prevent oxidative damage of cellular components. In plant cells, ascorbic acid (AsA) is a major antioxidant, which is a part of the AsA-glutathione (GSH) cycle^3^,^4^. In order to maintain the antioxidative capacity of AsA, rapid regeneration of AsA is regulated by dehydroascorbate reductase (DHAR) and monodehydroascorbate reductase (MDHAR)^3^,^5^,^6^. Activation of DHAR and MDHAR in these stress responses has been observed in different plants. In transgenic tobacco, overexpression of DHAR protected the plants against drought and ozone stress, whereas high expression of MDHAR increased their tolerance to osmotic stress^7^,^8^. In addition, an increase in MDHAR activity resulted in increased tolerance to chilling stress in tomato^9^,^10^.

The first reports of the crystal structure of DHAR of *Oryza sativa* (Os) L. *japonica* (OsDHAR) suggested that regeneration of AsA by DHAR proceeded via a ping-pong mechanism^11^. The OsDHAR structure revealed that the Cys20 residue was involved in the reduction of dehydroascorbate (DHA) to AsA. The catalytic cysteine residue is reduced back from sulphenic to sulphinic acid by catalytic cycling via glutaredoxin^11^. Therefore, regeneration of cysteine residue is required for continued AsA-GSH cycle. However, the catalytic cysteine residue can be irreversibly oxidized to sulphonic acid by hyperoxidation, which inactivates the enzyme. Thus, the role of MDHAR in the AsA regeneration cycle may be more efficient than that of DHAR under conditions of strong oxidative stress. This prediction was supported by the results of an *in vivo* assay using yeast cells in this study.

We report, for the first time, the crystal structure of MDHAR of *O. sativa* L. *japonica* (OsMDHAR) to understand its detailed mechanism. MDHAR reduces the monodehydroascorbate (MDHA) radical to AsA at the expense of an NAD(P)H molecule, with NADH being the preferred donor^5^,^12^. The deduced amino acid sequence of MDHAR indicates that it consists of flavin adenine dinucleotide (FAD) and the pyridine nucleotide-binding domain of flavoenzymes. Multiple sequence analysis of MDHAR shows that it shares high sequence similarity with bacterial flavoenzymes, such as iron-sulphur protein reductase, instead of plant flavoenzymes such as ferredoxin-NADP reductase^13^. Consistently, the overall structure of OsMDHAR is also similar to iron-sulphur protein reductase. However, OsMDHAR has a unique long loop that distinguishes it from the iron-sulphur reductase family.

In a previous kinetic study, Hossain *et al*. suggested that NAD(P)H reduces FAD in MDHAR. The reduced FAD successively donates electrons to MDHA, producing AsA^14^. The structural information from OsMDHAR, as well as the structures complexed with NAD, NADP, and both NAD and AsA, allows us to elucidate a detailed description of the catalytic mechanism at a molecular level. Structure-guided point mutations indicate that Arg320 and Tyr349 residues are critical for the activity of OsMDHAR. We determined the role of both these residues using structural analysis. Additionally, structural comparison of the OsMDHAR complex with NAD and NADP suggested that the preference for NADH as an electron donor might arise from additional hydrogen bonding near the AMP-ribose of NADH. The quaternary structure of OsMDHAR complexed with isoascorbic acid (ISD), an AsA analogue, suggests that the enzyme can accommodate multiple substrates^15^.

## Results and Discussion

### Overall structure of OsMDHAR

In order to elucidate the electron transfer mechanism employed by OsMDHAR, we determined the crystal structure of OsMDHAR and complexes of the enzyme with NAD and NADP. We also determined quaternary complex structures with FAD, NAD, and AsA or ISD. All four structures contain one molecule of FAD per monomer, which imparts a yellow colour to the crystals. Except for the crystals of apo OsMDHAR and the OsMDHAR-NAD complex, which have one molecule in the asymmetric unit, there were two molecules in the asymmetric unit of the crystals. In a size-exclusion chromatography experiment, the purified OsMDHAR protein was eluted as a monomer (Fig. S1), implying that it possibly functions as a monomer.

**Figure 1:**
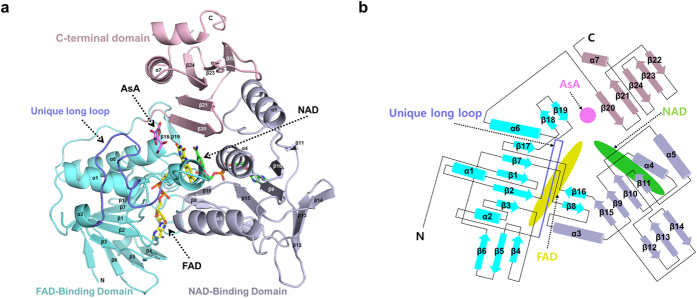
(**a**) Overall structure of OsMDHAR. The FAD, NAD, and C-terminal domains are coloured as cyan, light blue, and light pink, respectively. Specifically, the unique long loop is highlighted as blue. The bound FAD, NAD, and AsA molecules are presented as a stick model. (**b**) Topology diagram of OsMDHAR. The same colour scheme used for panel (**a**) was applied.

**Figure 2:**
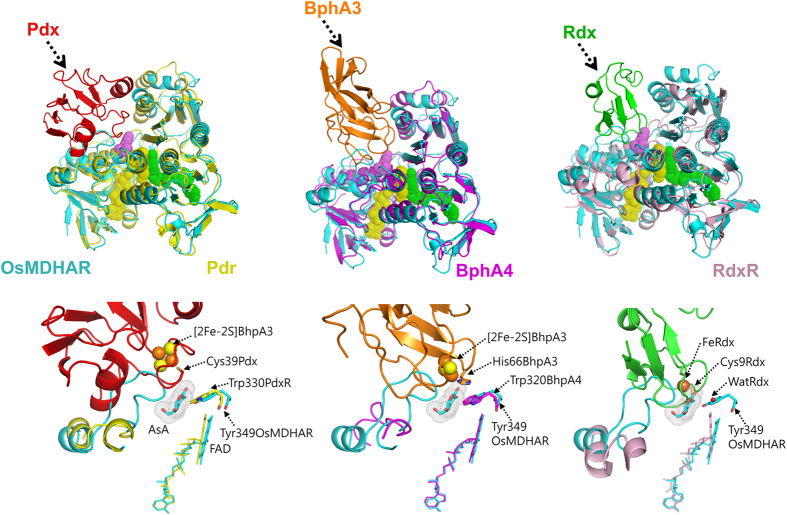
Structural superposition of OsMDHAR (cyan) with Pdr (PDB ID: 3LB8, yellow), BphA4 (PDB ID: 2YVJ, magenta), and RdxR (PDB ID: 2V3B, light pink) in a complex with Pdx (red), BphA3 (orange), and Rdx (green), respectively. FAD, NAD, and AsA are shown as spheres and coloured as yellow, green, and violet, respectively. Lower panel shows the close-up view of the substrate binding site. The bound AsA of OsMDHAR is shown as both a stick and a dots model. Iron clusters of Pdx, BphA3, and Rdx are shown as spheres. The residues that mediate electron transfer are shown as stick models. For clarity, only bound FAD are shown as stick models.

**Figure 3:**
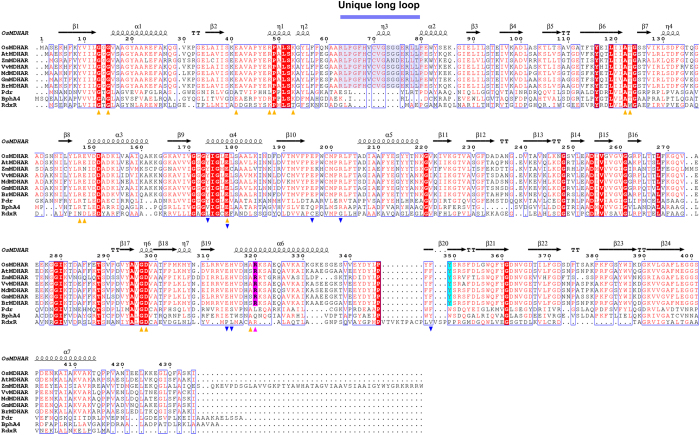
The multiple amino acid sequence alignment of the MDHAR homologues and iron-sulphur protein reductases. Secondary structure elements of OsMDHAR are labelled above the sequence. The catalytic Tyr residue that mediates electron transfer is highlighted by a cyan box and arrow. The residues comprising the unique long loop are also highlighted by blue boxes. The residues that participate in the FAD and NAD binding are indicated by orange triangles pointing up and blue triangles pointing down, respectively. The Arg320 residue forming hydrogen bonds with bound AsA is highlighted by a magenta box and triangle pointing up. Accession data for amino acid sequences are as follows: OsMDHAR from *O. sativa japonica* (UniProtKB code Q9XFZ3); AtMDHAR from *Arabidopsis thaliana* (UniProtKB code Q9LFA3); ZmMDHAR from *Zea mays* (UniProtKB code B8A028); VvMDHAR, from *Vitis vinifera* (UniProtKB code A5JPK7); MdMDHAR from *Malus domestica* (UniProtKB code C0LQ98); GmMDHAR from *Glycine max* (UniProtKB code A0A0R0I177); BrMDHAR from *Brassica rapa* (UniProtKB code Q93 × 74); Pdr, putidaredoxin reductase from *Pseudomonas putida* (UniProtKB code M5AXR7); BphA4, ferredoxin reductase from *Pseudomonas* sp. (UniProtKB code Q52437); RdxR, rubredoxin reductase from *P. aeruginosa* (UniProtKB code A0A0E1B6R8).

Structural comparison of OsMDHAR to other proteins, using the DALI server^16^, indicated that it has the same fold as that of putidaredoxin reductase (Pdr)^17^, biphenyl dioxygenase (BphA4)^18^ and rubredoxin reductase (RdxR)^19^. All these proteins consist of three domains: an NAD(P)-binding domain, an FAD-binding domain, and a C-terminal domain, as shown in Figs 1 and 2. The root mean square deviations (RMSD) after superposition of OsMDHAR with structures of Pdr, BphA4, and RdxR were 1.2–1.4 Å over 285, 255, and 208 Cα atoms, respectively. OsMDHAR also shares 45%, 41%, and 35% sequence similarity with Pdr, BphA4, and RdxR, respectively (Fig. 3). The bacterial reductases, Pdr, BphA4, and RdxR, transfer an electron from NAD(P)H via FAD to low molecular weight, iron-containing proteins: putidaredoxin (Pdx)^20^, ferredoxin (BphA3)^21^, and rubredoxin (Rdx)^19^, respectively. These redox partner proteins bind to the C-terminal domain of their corresponding reductases and mediate the electron transfer in a biological redox reaction. Although OsMDHAR also comprises three domains, similar to the above-mentioned reductases, it transfers an electron directly to its substrate, MDHA, which binds to its active site pocket. Superposition of the OsMDHAR structure with Pdr, BphA4, and RdxR structures complexed with their corresponding partner proteins (Pdx, BphA3, and Rdx) clearly shows the remarkable structural difference in terms of substrate binding site (Fig. 2)^19^,^20^,^21^. The superposed structures show that the unique long loop of 63–80 residues of OsMDHAR is adjacent to the iron cluster of Pdx, BphA3, and Rdx, and occupies a part of the partner proteins in the complexed structures. It is involved in the formation of a closed and narrow active site pocket with positively charged residues, which only accommodates small molecules such as MDHA. The amino acid sequence of MDHAR is relatively conserved within the MDHAR homologue proteins, as shown in the multiple sequence analysis in Fig. 3.

### FAD-binding site

**Figure 4:**
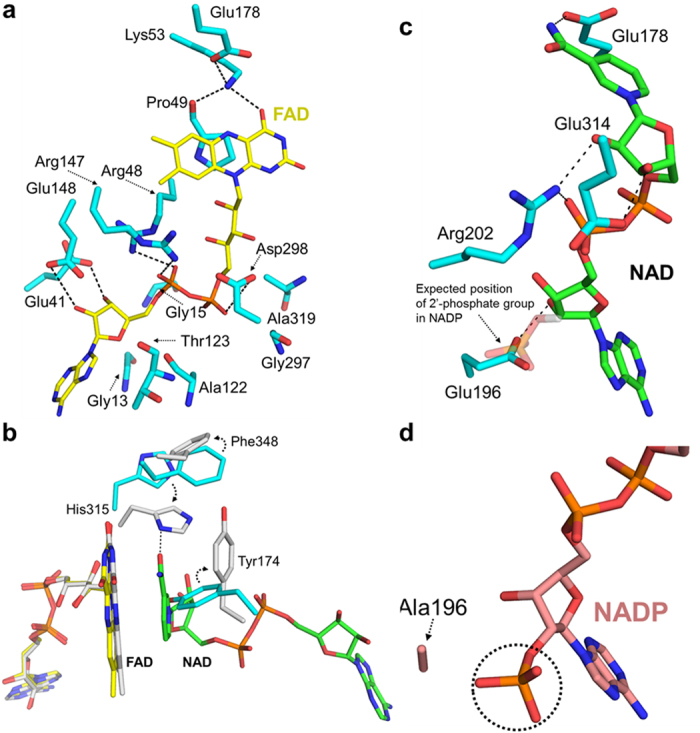
(**a**) Close-up view of the FAD binding site. The residues that participate in the hydrogen bond and van der Waals interactions are shown as stick model and coloured cyan. The bound FAD is coloured yellow. (**b**) Superposition of the FAD and NAD binding site of apo with the NAD-complex form of OsMDHAR (cyan and grey, respectively). The FAD and NAD of apo structure are coloured yellow and green, respectively, and only FAD of NAD-complex structure is shown as a stick model and coloured grey. The side chains are shown for the positions of the ‘Tyr174’, ‘His315’, and ‘Phe348’ residues, which display the largest conformational changes upon NAD binding. (**c**) Close-up view of the NAD binding site. The residues participating in the hydrogen bond are shown as a stick model. Note that the predicted 2′-phosphate group of NADP overlaps with Glu196. (**d**) Close-up view near 2′-phosphate group in NADP bound structure. For clarity, only Ala196 is shown as a stick model. The Glu196 of OsMDHAR-NAD is shown as a stick model and coloured cyan. The 2′-phosphoryl group of NADP is highlighted by circle. All of the hydrogen bonding is within a distance of 3.5 Å.

The FAD-binding domain of OsMDHAR adopts a typical α/β fold consisting of two antiparallel β-sheets and one parallel β-sheet surrounded by four α-helices, as shown in Fig. 1. The FAD is bound to a crevice on the FAD-binding domain of the protein via hydrogen bonds and van der Waals interactions, as shown in Fig. 4a. The residues participating in the van der Waals interactions with FAD are Gly13, Gly15, Ala122, Thr123, Gly297, and Ala319. Most of them are located at the bottom of the crevice and are highly conserved. Lys53, salt-bridged with Glu178, participates in hydrogen bond formation with O4 of FAD, which is also hydrogen bonded with the carbonyl oxygen of Pro49. Both of the residues, Glu178 and Pro49, are highly conserved, and the hydrogen bond network among Lys53, Glu178, and FAD is commonly observed in other enzymes such as glutathione reductase and trypanothione reductase^17^,^18^,^22^,^23^. The hydrogen bond network indicates that no residues are available for proton transfer to the N5 atom of FAD, thus preventing stabilization of flavin semiquionone by OsMDHAR^17^,^24^.

The conserved residue Asp298 forms a hydrogen bond with O3’ of the ribitol moiety and the phosphate group is stabilized by hydrogen-bond interactions with Arg48 and Arg147. The Glu41 and Glu148 residues are hydrogen bonded with the O3B and O2B atoms of the adenosine moiety of FAD, respectively. Among those residues, Arg48 helps FAD binding by partially compensating for the negative charge of one of the phosphate groups of FAD^22^.

### Conformational changes induced by binding of NAD

The NAD(P) binding site consists of residues 125 to 264. The NAD-binding domain in OsMDHAR consists of antiparallel β-sheets (β8, β12, β13, β14, and β16) and parallel β-sheets (β9, β10, β11, and β15) surrounded by three α-helices (α3, α4, and α5) (Fig. 1b). Reduction of the crystals by binding of NAD quenches the intrinsic yellow colour of the isoalloxazine of FAD, resulting in colourless crystals. The binding of NAD led to a butterfly-like movement of the isoalloxazine ring of FAD as shown in Fig. S2, which was previously observed in the structure of BphA4^21^. This movement is most likely induced by a change in the redox state of FAD^21^. We observed two different redox states of FAD, namely oxidized and reduced forms, by measuring the UV-visible spectrum of the OsMDHAR protein (Fig. S2). We observed significant conformational changes only in the residues around FAD and NAD binding sites, on comparing the overall structure of OsMDHAR-NAD complex to the NAD-free form. The RMSD between equivalent Cα atoms of the NAD-free and OsMDHAR-NAD complex was 0.61 Å. NAD binding accompanied a large conformational shift in Tyr174, His315, and Phe348 residues. Tyr174, which points toward the NAD binding site in the NAD-free structure, is moved away from the nicotinamide ring upon NAD binding due to steric hindrance. As a result, the nicotinamide ring is sandwiched between the isoalloxazine ring of FAD and the side chain of Tyr174. Then, the neighbouring Phe348 residue shifts outward to avoid steric conflict with Tyr174. In contrast, His315 is oriented towards the NAD binding site and hydrogen bonded with O7N atom of the bound NAD. The above three residues are only conserved through MDHAR homologues. Thus, this conformational change, induced by NAD binding, has not been observed in other flavoprotein-NAD complexes.

In addition to the van der Waals contacts around the nicotinamide moiety, a conserved Glu178 residue participates in hydrogen bond interaction with the nicotinamide ring, as shown in Fig. 4c. The OE2 atom of Glu178 forms a hydrogen bond with the N7N atom of the nicotinamide ring. The NH2 atom of Arg202 forms hydrogen bond with the O2D of the ribose and the O2A of the phosphate group, respectively. An additional hydrogen bond was formed between the OE1 atom of Glu314 and the O3D atom of ribose. Specifically, in the OsMDHAR structure, an extra hydrogen bond was observed between the O2B of the adenosine ring and Glu196.

### Preference for NAD over NADP

**Figure 5:**
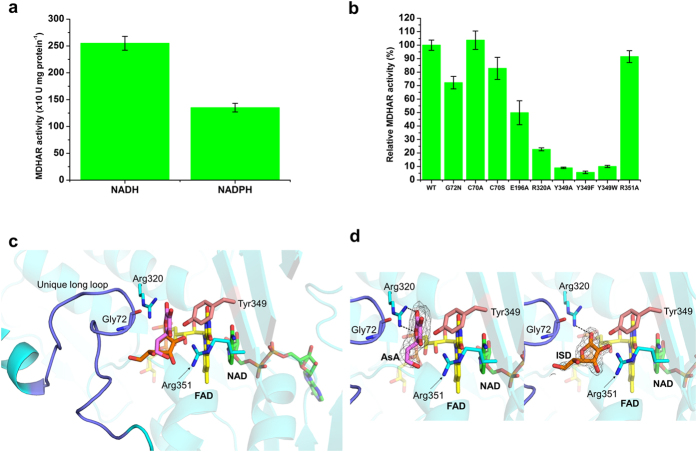
(**a**) MDHAR activity was measured using NADH or NADPH as an electron donor. The activity decreases when NADPH is used as an electron donor. (**b**) The MDHAR activity of the mutant was compared with the wild-type (WT) enzyme. The E196A, R320A, and Y349X mutants retain less than 50% of WT enzyme activity. (**c**) The substrate binding site of OsMDHAR. The bound AsA and ISD are shown as a stick model and coloured violet and orange, respectively. The bound FAD and NAD molecules are also shown as a stick model and coloured yellow and green, respectively. The loop between residues 63 and 80 is highlighted as blue. The residues which are located around the binding pocket are shown as a stick model. For clarity, Phe349 of the Y349F structure is mutated and shown as Tyr349 and coloured salmon. (**d**) Close-up view of the substrate binding site. The electron density map of AsA and ISD are presented. The map as calculated with (2|F_*o*_|−|F_*c*_|) and contoured at 1.5 σ. Note that Arg320 is hydrogen bonded with both the AsA and ISD complex structure.

Previous reports have demonstrated that reduction of Pdr by NADPH was three orders of magnitude slower than with NADH^25^. This is possibly due to hydrogen bonding of an additional Glu186 with the ADP-ribose of NAD. In addition, the crystal structure of Pdr indicated that the relatively long and flexible Glu186 of Pdr might disrupt the binding of the 2′-phosphate group of NADP, thereby increasing selectivity of the enzyme for NAD^17^. However, because the structure of Pdr complexed with NAD or NADP is not available, the above speculation is not confirmed. The biochemical data shown in Fig. 5a indicates that OsMDHAR also prefers NADH to NADPH. We postulated that, similar to Pdr, the preference for NADH in the OsMDHAR structure may arise from an additional hydrogen bond with the ADP-ribose of NAD. Our speculation was confirmed from the structure of the OsMDHAR-NAD complex, where we observed hydrogen bonding between Glu196 and the O2B molecule in the adenosine ring of NAD (Fig. 4c). Abolishing the hydrogen-bonding interaction by introducing an Ala196 mutation (E196A) reduced the enzyme activity, as shown in Fig. 5b. When NADP was docked into the NAD binding pocket of OsMDHAR, the Glu196 residue occupied the site of the 2′-phosphate group of NADP (Fig. 4c). Figure 3 shows the sequence alignment of OsMDHAR that revealed Glu196 to be a highly conserved residue throughout MDHAR homologues. For more detailed information, we attempted to determine the structure of the enzyme in complex with NADP. We prepared an E196A mutant protein, which facilitates the binding of NADP by eliminating the carboxylic acid group. Unexpectedly, the structure of the E196A mutant-NADP complex revealed that the 2′-phosphate group of NADP was not located in the position of the carboxylic acid group of Glu196.

To test whether the long and flexible Glu196 interferes with the binding of NADPH, we measured the binding affinity of wild type (WT) and E196A for NADH and NADPH using isothermal titration calorimetry (ITC). ITC experiments clearly showed an increase in the affinity of OsMDHAR toward NADP on removing the carboxylate group of Glu196 (Fig. S3). ITC data showed that the binding affinity of WT for NAD was 20-fold higher than that of WT for NADP. On the other hand, E196A had only about 3 times higher affinity for NAD than that for NADP. Although there is no difference between WT and E196A in NAD binding affinity, E196A showed a 16-fold increase in affinity for NADP compared to the WT. Therefore, together from the structural studies and ITC experiments, we hypothesized that Glu196 has an important role in the NAD selectivity of the OsMDHAR.

### Substrate binding site

Some of the bacterial reductases like Pdr, BphA4, and RdxR, which have a high structural similarity with OsMDHAR, interact with their redox partner proteins to form redox-protein complexes. In the electron transfer pathway from structures of the redox-protein complexes, the conserved Trp residues in Pdr and BphA4 are able to mediate electron transfer from FAD to the [2Fe-2S] cluster, whereas RdxR has a bridging catalytic water molecule instead of a catalytic residue for the same. In OsMDHAR, Tyr349 is a completely conserved residue in the homologues of MDHAR, which structurally corresponds to the catalytic residues in similar enzymes (Figs 2 and 3). Mutation of this Tyr349 residue to Ala, Phe, and Trp abolishes the enzyme activity, as shown in Fig. 5b. Based on these results, we speculate that Tyr349 is a catalytic residue of OsMDHAR.

**Figure 6:**
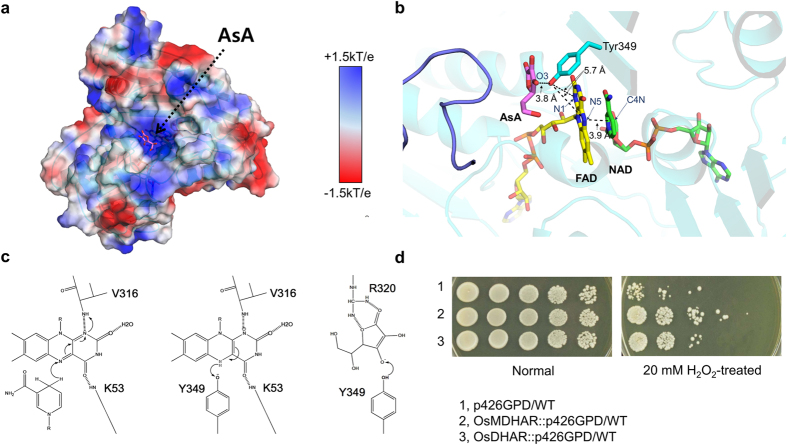
(**a**) Electrostatic surface potential of OsMDHAR shows that the surface of the AsA binding site is highly basic. (**b**) Close-up view of the electron transfer pathway from NAD to AsA via FAD by flanking Tyr349. (**c**) Proposed electron transfer mechanism of OsMDHAR. Left, reduction process of the first FMN by the stacked NADH; middle, reduction process of the Tyr349 by the FMN; right, reduction process of the bound substrate (MDHA) by the Tyr349. The backbone amide nitrogen atom of Val316 is within hydrogen bonding distance (3.15 Å), which can either stabilize the negative charge of the semiquinone from or provide a proton to it. The arrows indicate the movement of an electron. (**d**) Comparison of adaptation ability to oxidative stress in *OsDHAR*- and *OsMDHAR*-expressing yeast cells. Mid-log phase yeast cells were exposed to 20 mM H_2_O_2_ for 1 h, serially diluted with YPD medium, and spotted onto a YPD agar plate. 1, wild-type (WT) cells with an empty vector alone; 2, WT cells expressing *OsMDHAR*; 3, WT cells expressing *OsDHAR*.

To elucidate the detailed mechanism of the electron pathway adopted by OsMDHAR, we co-crystallized OsMDHAR with AsA, which is the final product of the enzyme, in the presence of NAD. We determined the structure of the catalytically compromised Y349F mutant in complex with AsA. Superposition of the Tyr349 and Phe349 of apo and mutant structure indicates that there is no conformational change of Phe349 (Fig. S4a). In both apo and NADH complex structure, Tyr349 formed hydrogen bonds with two water molecules, whose position was corresponded to the bound substrate ligand in the AsA complex structure (Fig. S4b). This suggests that hydrogen bonding with water molecules might limit the flexibility of Tyr349, and ligand binding is attained by the replacement of the hydrogen-bonded water molecules. In case of other enzyme-protein complexes, such as the BphA4-BphA3 complex, binding of the redox partner protein induced rotational movement of the NAD-binding domain and C-terminal domain^21^. However, we did not observe any large conformational changes in the structure of the OsMDHAR-NAD-AsA complex. The active site of OsMDHAR consists of highly positively charged electrostatic potential pockets, which easily bind to the negatively charged AsA, as shown in Fig. 6a. Structural studies revealed three highly conserved residues, G72, R320, and R351, positioned closed to the AsA molecule (Figs 3 and 5c). Of these residues, Arg320 forms a hydrogen bond with AsA bound to the structure (Fig. 5d). In addition, in the mutational analysis, only R320A had significantly reduced activity (Fig. 5b). Therefore, we suggest that R320 is important for substrate binding.

### Electron transfer mechanism

The structural and mutagenesis studies suggest that the electron is transferred from the flavin N atom to the FAD flanking Tyr349, which subdivides the distance between FAD and AsA into two shorter segments of 5.7 and 3.8 Å, and then directly to the substrate, MDHA (Fig. 6b). As shown in Fig. 6c, initially, two electrons are transferred from NAD(P)H in the hydride transfer to FAD in OsMDHAR. During the oxidative reaction, two electrons are sequentially transferred from FADH_2_ to Tyr349, resulting in a flavin semiquinone reaction intermediate. The electrons are then transferred from the Tyr349 to the MDHA radical, generating two molecules of AsA.

MDHAR uses a catalytic tyrosine residue to regenerate AsA unlike DHAR, which uses a catalytic cysteine residue that is inactivated by hyperoxidation. Mutation of Cys70, which is the only Cys residue in OsMDHAR, had no significant effect on enzyme activity (Fig. 5b). Based on this observation, we postulated that MDHAR plays a major role in AsA recycling. To verify our hypothesis, we cultured three types of yeast cells harbouring different genes: the OsDHAR gene, the OsMDHAR gene, or an empty vector. Cells expressing either OsMDHAR or OsDHAR were more resistant under H_2_O_2_-induced oxidative stress compared to WT cells that harbour an empty vector (Fig. 6d). However, yeast cells overexpressing MDHAR were more tolerant to oxidative stress than those expressing DHAR, verifying the predominant role of MDHAR in oxidative stress (Fig. 6d).

During data interpretation, we noticed that although most eukaryotic organisms, including plants, produce L-ascorbic acid, yeast cells instead produce D-erythroascorbic acid, a five-carbon analogue of L-ascorbic acid^26^,^27^. The fact that both enzymes are functional in our *in vivo* assay implies that MDHAR and DHAR can use an alternate substrate analogue such as D-erythroascorbic acid. For structural confirmation of binding of the enzyme to an AsA analogue, we co-crystallized OsMDHAR bound to NADH and isoascorbic acid (ISD), as D-erythroascorbic acid is not commercially available. We observed an interpretable electron density map, suitable for ISD fitting. Superimposed structures of OsMDHAR-AsA and OsMDHAR-ISD complexes showed distinct binding conformations for both substrate ligands (Fig. 5c,d). In detail, ISD did not fit perfectly into the binding site of AsA. The binding site of ISD had more exposed surface area. In addition, we observed clear electron density for the di-hydroxyfuranone part of ISD, but not its di-hydroxyethyl part. This suggests that ISD may bind more loosely to the enzyme than AsA. Nevertheless, ISD is hydrogen bonded to the conserved Arg320, which is within an accessible distance for electron transfer from the Tyr349 residue. Kinetic analysis of OsMDHAR for AsA and ISD revealed that the catalytic Vmax values were 37.5 ± 2.5 and 32.6 ± 2.7, respectively, whereas the Km values were almost identical (Fig. S5). The Arg320 residue forms hydrogen bonds with both AsA and ISD in the complex structures. This is evidence that Arg320 plays an important role for AsA as well as ISD despite their dissimilar binding modes. We confirmed this through an enzyme activity assay using an R320A mutant which retained only 20% activity compared to WT. The catalytic hydroxyl group of the active site Y349 residue was positioned within reasonable distance to be able to transfer electrons to O3 of AsA or ISD. The Y349X mutants also displayed almost no activity compared to WT enzyme (Fig. 5). Arg320 and Tyr349 are highly conserved in other MDHAR homologues (Fig. 3). Therefore, we concluded that Arg320 actually has a substrate-binding role for both substrates and that the Tyr349 residue mediates electron transfer toward AsA or ISD.

We also confirmed the physiological role of AsA and ISD against oxidative stress in yeast mutants. The enzymatic activity of OsMDHAR, produced from yeast cells, was consistent with both AsA and ISD (Fig. S6a). The antioxidant effect and regeneration of AsA and ISD in yeast cells was confirmed using yeast mutant cells (*ara2*∆), which lacked the *ARA2* gene coding D-erythroascorbic acid biosynthesis. Exogenous AsA and ISD in the yeast mutant cells recovered rapidly, when challenged with H_2_O_2,_ compared to the cells lacking antioxidant molecule supplementation. As shown in Fig. S6b, there is a subtle difference between the adaptative abilities of AsA and ISD. The yeast mutant cells expressing OsMDHAR were more tolerant to oxidative stress, compared to the control cells without OsMDHAR. These results suggest that both antioxidants, AsA and ISD, are able to substitute D-erythroascorbic acid, and their reduced forms are regenerated by MDHAR activity in yeast cells. Given the structural and functional analysis, we conclude that the binding site of OsMDHAR may be able to accommodate variable substrate compositions including MDHA.

## Methods

### Cloning, purification, and preparation of mutants

The cloning of the *OsMDHAR* gene was described in the previous report^28^. In brief, *OsMDHAR* was amplified from cDNA of *O. sativa* L. *japonica* by PCR and PCR products were integrated into the pKM260 vector (Euroscarf, Germany). The recombinant plasmid was transformed into *Escherichia coli* strain NiCo21 (DE3) cells. For purification, cells were resuspended in buffer A (20 mM Tris-HCl, pH 8.0, 200 mM NaCl, and 5 mM imidazole) and lysed by sonication. The lysate was centrifuged and loaded onto a HisTrap HP column (GE Healthcare, USA). The column was washed with wash buffer (20 mM Tris-HCl, pH 8.0, 200 mM NaCl, and 20 mM imidazole). Then, protein was eluted with buffer B (20 mM Tris-HCl, pH 8.0, 200 mM NaCl, and 300 mM imidazole). The protein was purified to its final state by gel filtration on a HiLoad 16/60 Superdex 200 column (GE Healthcare, USA) that had previously been equilibrated with 20 mM Tris-HCl, pH 8.0 and 200 mM NaCl. The protein was finally concentrated to 20 mg mL^−1^. Mutant genes of OsMDHAR (C70A, C70S, G72N, E196A, R320A, Y349A, Y349F, Y349W, and R351A) were prepared by site-directed mutagenesis using PCR. All the mutant proteins were expressed in *E. coli* NiCo21 (DE3) cells^29^ and subsequent purification of mutants OsMDHAR protein was identical as described above for the wild-type protein^28^.

### Enzymatic activity

MDHAR activity was assayed spectrophotometrically (UV-1650PC; Shimadzu Corp., Kyoto, Japan). The assay was performed at 25 °C with a reaction mixture (1 mL) containing 50 mM potassium phosphate, pH 7.2, 0.25 mM NADH or NADPH, 2 mM AsA, 1 unit AsA oxidase (Sigma-Aldrich, USA), and purified protein (10 μg). The absorbance was measured at 340 nm by monitoring NAD(P)H oxidation, and the activity was calculated using an absorbance coefficient of 6.2 mM^−1^cm^−1^. One unit is the amount of enzyme that oxidizes 1 nmol of NADH per min at 25 °C. Enzyme activity was represented relative to WT proteins, which was defined as 100%. The enzymatic assay was independently performed at least 3 times and results are expressed as the mean ± standard deviation (SD).

### Crystallization and structure refinement

The crystallization of apo OsMDHAR was described in the previous report^28^. Briefly, apo OsMDHAR crystals were obtained under conditions consisting of 100 mM Tris–HCl, pH 8.0, 200 mM lithium sulphate, 30% (w/v) PEG 5000 MME by the hanging-drop vapour-diffusion method. The initial crystal screening of OsMDHAR mutant crystals (E196A and Y349F) was also performed with a protein of 15 mg mL^−1^, and crystals appeared under conditions consisting of 100 mM Tris-HCl, pH 8.5, 200 mM ammonium acetate, 25% (w/v) PEG 3350. For complex crystals with NAD (OsMDHAR-NAD), apo crystals were soaked with 10 mM NADH for 10 min until the crystals became transparent. For co-crystallization with NADP (OsMDHAR-NADP), 50 mM of NADPH was added to the crystals of E196A and incubated for approximately 30 min until the crystals changed from yellow to transparent. For the complex crystals with NAD-AsA or ISD (OsMDHAR-NAD-AsA or ISD), crystals of Y349F were first soaked with 10 mM NADH and incubated for about 30 min until the yellow colour of the crystals disappeared. Then, additional soaking of crystals with 10 mM concentrations of AsA or ISD was carried out for 30 min.

In a previous paper, the data collection and structural refinement of apo OsMDHAR was described^28^. In the case of complex crystals, Perfluoropolyether Cryo Oil (Hampton Research, USA) was introduced as a cryoprotectant. X-ray diffraction data for complex crystals with NAD were collected at a resolution of 2.0 Å using an ADSC Quantum 315 CCD detector on beamline 5C at the Pohang Light Source, Pohang, South Korea. X-ray diffraction data for complex crystals with NADP were collected at a resolution of 1.8 Å using an ADSC Quantum 4 CCD detector on beamline 7A at the Pohang Light Source, Pohang, South Korea. X-ray diffraction data for complex crystals with AsA were collected at 2.3 Å using an ADSC Quantum 210 CCD detector on beamline BL44XU at the SPring-8, Hyogo, Japan. X-ray diffraction data for complex crystals with ISD were collected at 1.9 Å using an ADSC Quantum 315 CCD detector on beamline 5C at the Pohang Light Source, Pohang, South Korea. All data sets were indexed, processed and scaled using the HKL-2000 software package^30^.

The crystal structure of apo OsMDHAR was solved by the molecular replacement (MR) method, using the *MOLREP* program in the *CCP4* package^31^ with biphenyl dioxygenase BphA4 (PDB ID: 4H4Q) as a search model^28^. Then, the refinement was performed using *Refmac5*^32^ and the model was rebuilt with the *COOT* program^33^. Subsequently, the resulting structure of apo OsMDHAR was used as search model for the complex structures in NAD, NADP, AsA, and ISD. The stereochemical qualities for all of the final models as assessed by *MolProbity* were excellent^34^. The refinement statistics are summarized in Table 1. All structural figures were generated using the *PyMOL* program^35^.

### Protein data bank accession number

The coordinates of the structures together with the structure factors have been deposited in the Protein Data Bank (http://www.rcsb.org/pdb) with accession codes of 5JCI, 5JCK, 5JCL, 5JCM, and 5JCN.

### Stress response in *OsMDHAR*- and *OsDHAR*-expressing yeast cells

cDNA containing the open reading frame (ORF) of OsMDHAR and OsDHAR was subcloned into the yeast expression vector p426GPD (yeast 2 μ expression vector with URA3 marker; GenBank Accession No. DQ019861), which allows constitutive expression of a target gene under the control of the yeast *GPD* (glyceraldehyde-3-phosphate dehydrogenase) promoter^36^. To measure cell viability, cells (1 × 10^6 ^cells per mL) grown at 28 °C overnight were inoculated in YPD medium (1% yeast extract, 2% peptone and 2% dextrose) and cultured for 8 h. Mid-log phase cells (OD_600_ ≈ 3.0) were exposed to 20 mM H_2_O_2_ for 1 h at 28 °C and serially diluted (10^0^ to 10^−4^) with YPD medium. Five microliters of diluted solutions were spotted onto YPD agar plates and incubated for 2–3 days at 28 °C. The results of the spotting assay are representative of at least 2 independent experiments performed under identical conditions.

## Additional Information

**How to cite this article**: Park, A. K. *et al*. Structure and catalytic mechanism of monodehydroascorbate reductase, MDHAR, from *Oryza sativa* L. *japonica*. *Sci. Rep.* **6**, 33903; doi: 10.1038/srep33903 (2016).

## Supplementary Material

Supplementary Information

## Figures and Tables

**Table 1:** Data collection and refinement statistics.

	WT	WT_NAD	E196A	Y349F	Y349F
			_NADP	_NAD	_NAD
				_AsA	_ISD
PDB ID	5JCI	5JCK	5JCL	5JCN	5JCM
Data collection					
Space group	*P*4_1_2_1_2	*P*4_1_2_1_2	*P*2_1_2_1_2_1_	*P*2_1_2_1_2_1_	*P*2_1_2_1_2_1_
Cell parameters (Å)	a = b = 81.3,	a = b = 81.5,	a = 78.2, b = 85.6,	a = 79.6, b = 85.1	a = 78.4, b = 85.1,
	c = 120.7,	c = 121.4,	c = 131.4,	c = 133.4,	c = 131.8,
	α = β = γ = 90°	α = β = γ = 90°	α = β = γ = 90°	α = β = γ = 90°	α = β = γ = 90°
Resolution (Å)	50.0–1.7 (1.73–1.7)	50.0–2.0 (2.03–2.0)	50.0–1.8 (1.83–1.8)	50.0–2.3 (2.34–2.3)	50.0–1.9 (1.93–1.9)
No. of unique reflections	85440	53422	82522	41172	69512
*R*_merge_ (%)	6.3 (29.5)	14.5 (35.5)	7.2 (69.7)	7.3 (50.2)	8.2 (65.6)
I/σ(I)	41.4 (7.3)	25.7 (7.7)	56.6 (4.5)	56.0 (2.3)	52.4 (3.7)
Completeness (%)	99.2 (100)	99.7 (100)	99.7 (100)	99.5 (100)	99.3 (98.8)
Redundancy	4.8 (4.7)	14.1 (14.4)	13.2 (13.4)	13.9 (14.9)	14.1 (14.9)
Refinement					
*R*_*f*actor_ (%)	17.3	15.9	18.1	20.6	17.4
*R*_*free*_ (%)	20.4	20.2	23.1	26.4	22.4
RMS deviations					
Bond lengths (Å)	0.009	0.01	0.019	0.019	0.02
Bond angles (°)	1.43	1.722	2.125	2.038	2.225
Average B-factor (Å^2^)					
Protein	21.8	19.7	37.3	54.4	39.3
Ligand	14	12	27.1	66.6	34.3
Water	33.8	29.9	45.3	71.5	44.7

The values in parentheses refer to the highest resolution shell.

*R*_factor_ = Σ||*F*_o_| − |*F*c||/Σ|*F*_o_|. 5% of reflections have been used for *R*_free_ calculations.
