# Supplementary material for: Structure and catalytic mechanism of monodehydroascorbate reductase, MDHAR, from Oryza sativa L. japonica
Source: Sci Rep. 2016 Sep 22;6:33903. doi: 10.1038/srep33903 (PMC5031999; doi:10.1038/srep33903)
Supplement: Supplementary Information [file srep33903-s1.doc]

**Supplementary Information**

**Structure and catalytic mechanism of monodehydroascorbate reductase, MDHAR, from *Oryza sativa* L. *japonica***

Ae Kyung Park^1,6^, Il-Sup Kim^2,6^, Hackwon Do^1,6^, Byung Wook Jeon^1^, Chang Woo Lee^1,3^, Soo Jung Roh^1^, Seung Chul Shin^1^, Hyun Park^1,3^, Young-Saeng Kim^4^, Yul-Ho Kim^5^, Ho-Sung Yoon^2*^, Jun Hyuck Lee^1,3*^, Han-Woo Kim^1,3*^

^1^Division of Polar Life Sciences, Korea Polar Research Institute, Incheon 21990, Republic of Korea

^2^School of Life Sciences, BK21 Plus KNU Creative BioResearch Group, Kyungpook National University, Daegu 41566, Republic of Korea

^3^Department of Polar Sciences, University of Science and Technology, Incheon 21990, Republic of Korea

^4^Research Institute for Ulleung-do & Dok-do, Kyungpook National University, Daegu 41566, Republic of Korea

^5^Highland Agriculture Research Institute, National Institute of Crop Science, Rural Development Administration, Pyeongchang 25342, Republic of Korea

^6^A.K.P, I-.S.K and H.D. equally contributed to this work.

^*^To whom correspondence should be addressed:

Dr. Ho-Sung Yoon, Department of Biology, College of Natural Sciences, Kyungpook National University, Daegu 41566, Republic of Korea; Tel: +82-53-950-5348; Fax: +82-53-953-3066, E-mail: [hsy@knu.ac.kr](mailto:hsy@knu.ac.kr)

Dr. Jun Hyuck Lee, Division of Polar Life Sciences, Korea Polar Research Institute, Incheon 21990, Republic of Korea; Tel: +82-32-760-5555; Fax: +82-32-760-5509; E-mail: [junhyucklee@kopri.re.kr](mailto:junhyucklee@kopri.re.kr)

Dr. Han-Woo Kim, Division of Polar Life Sciences, Korea Polar Research Institute, Incheon 21990, Republic of Korea, Tel: +82-32-760-5526; Fax: +82-32-760-5509; E-mail: [hwkim@kopri.re.kr](mailto:hwkim@kopri.re.kr)


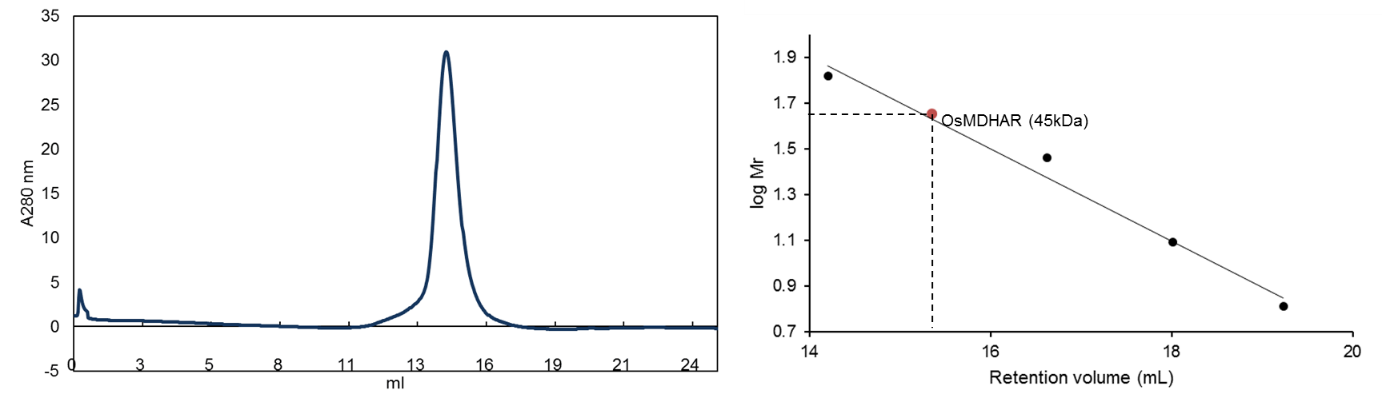


Figure S1. Analytical size exclusion chromatography of OsMDHAR (residues 1–435; calculated molecular weight of 45 kDa including tags for the polypeptide chain). The molecular mass of recombinant OsMDHAR was determined by analysis of the elution of standard proteins from a Superdex 200 10/300 GL column (GE Healthcare, USA). Chromatograms of OsMDHAR showed that OsMDHAR exists as a monomer in solution. The column was calibrated with molecular mass standards: albumin (66 kDa), carbonic anhydrate (29 kDa), cytochrome C (12.4 kDa), aprotinin (6.5 kDa).


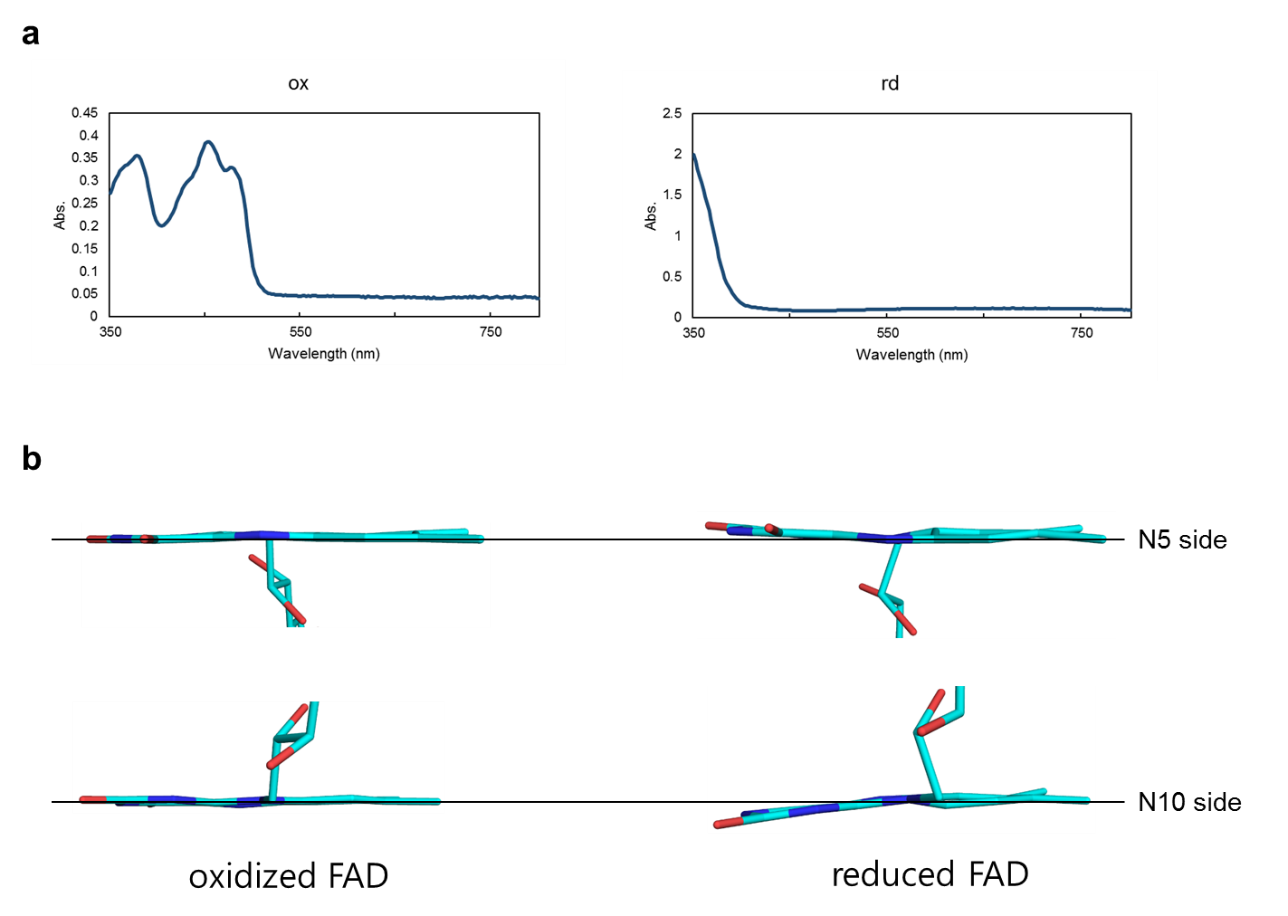


Figure S2. UV-visible spectrum (a), and conformation (b) of the FAD isoalloxazine ring in the structure of OsMDHAR. The UV-visible spectrum of OsMDHAR was monitored in the absence (oxidized, ox) and the presence of NADH (reduced, rd) in the solution state. The structure of apo- (oxidized form) and NADH-bound OsMDHAR (reduced form) was refined using no restraint in the Refmac program. The structural conformations of FAD in two states viewed from the N5 and N10 sides are also shown as stick models.


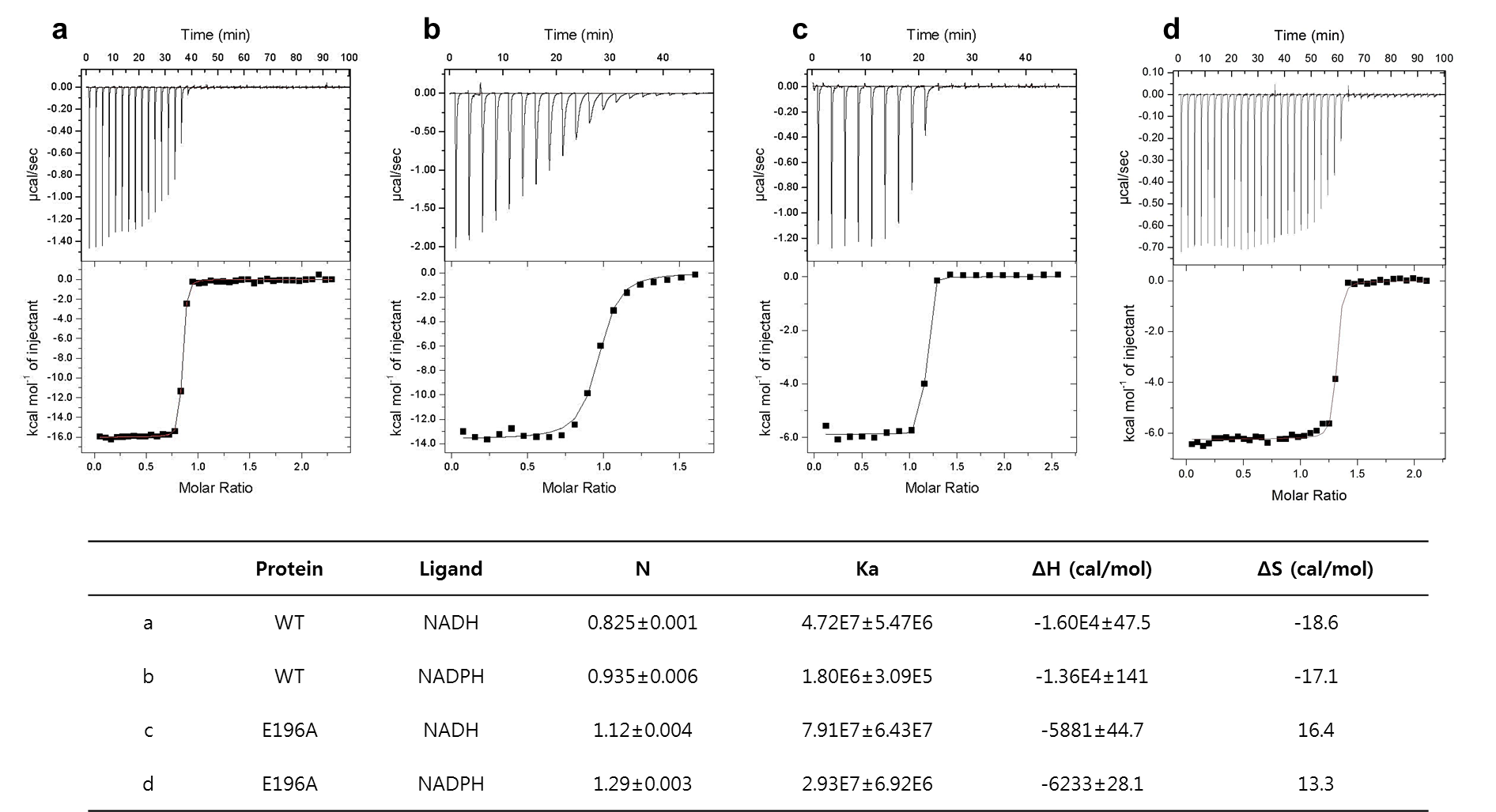


Figure S3. ITC analysis of cofactor binding affinity. Isothermal titration calorimetry profiles for NADH binding to wild-type OsMDHAR (WT) (a), NADPH binding to WT (b), NADH binding to E196A mutant (c), and NADPH binding to E196A (d). Raw injection heats (expressed as differential power) are shown in the top panels and the corresponding specific binding isotherms (calculated from the integrated injection heats and normalized to moles of injectant) are shown in the bottom panels.


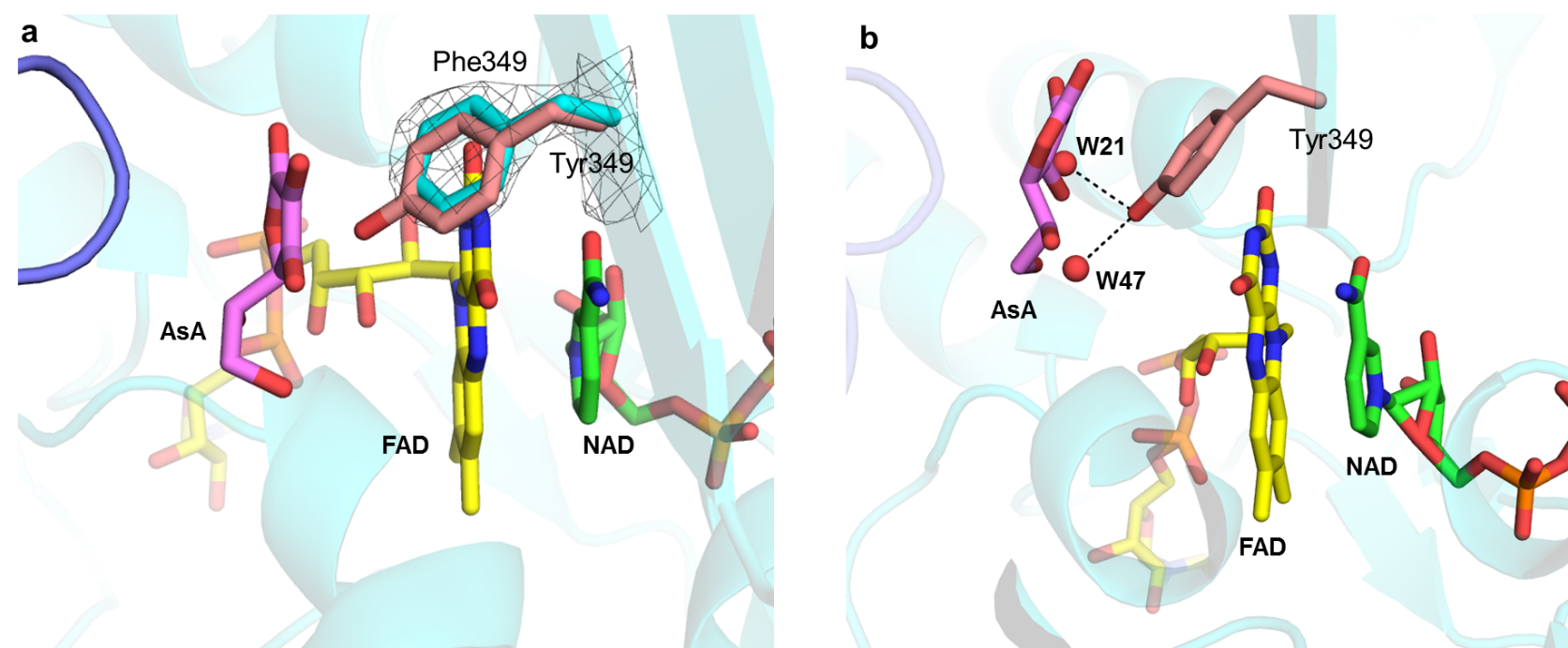


Figure S4. Superposition of apo and OsMDHAR-AsA complex structures of OsMDHAR. (a) Note that there is no conformational change of Phe349 in the mutant structure. The electron density map of Phe349 is presented. The map is calculated with (2|Fo|-|Fc|) and contoured at 1.5 σ. (b) Tyr349 in the apo structure and the OsMDHAR-NAD complex structure forms hydrogen bonds with two water molecules, which are replaced by an AsA molecule in the OsMDHAR-AsA complex structure.


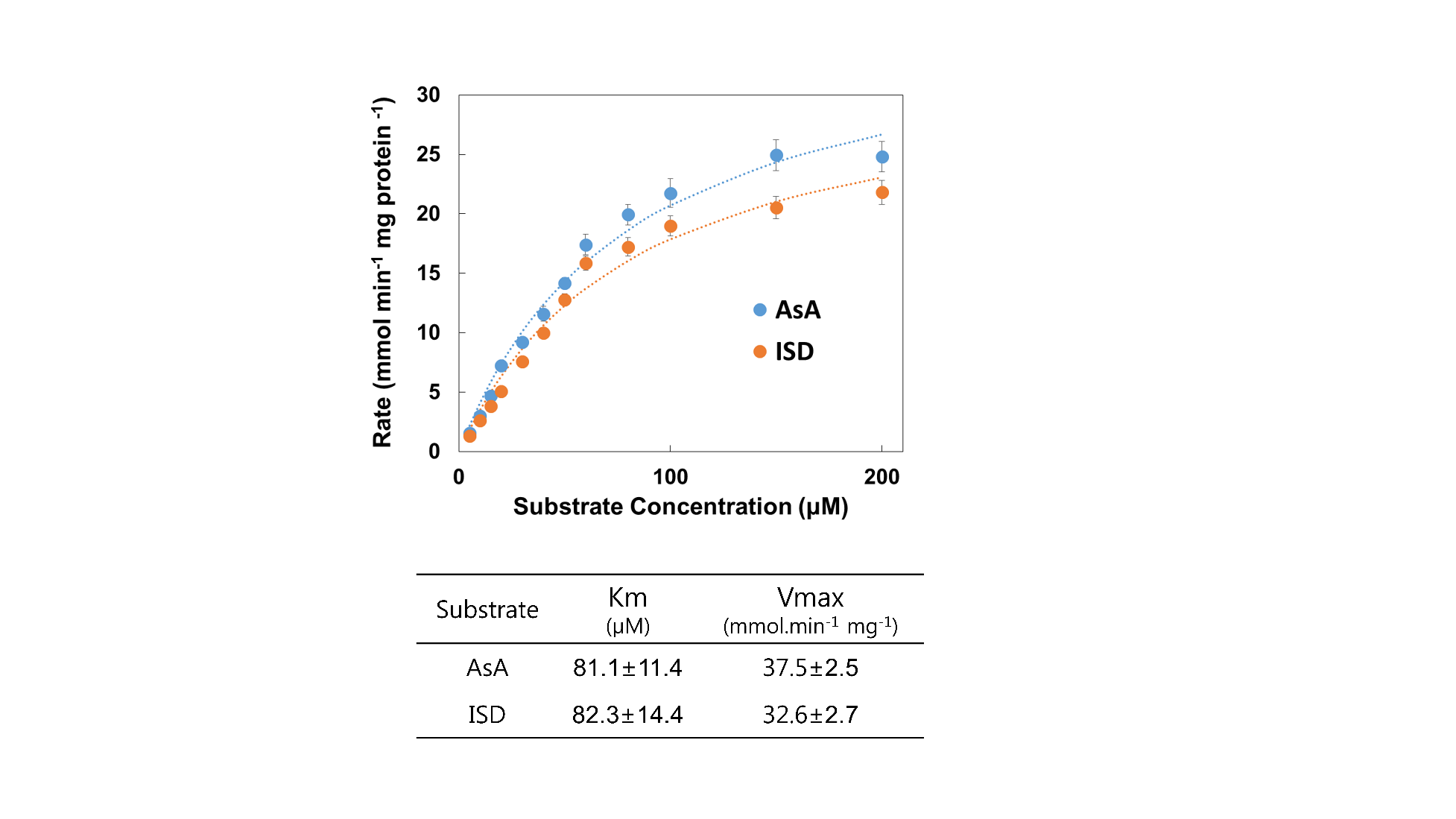


Figure S5. Michaelis-Menten plots of purified OsMDHAR and kinetic analysis of the substrates, AsA and ISD. The assay was performed at 25 °C with a reaction mixture (1 mL) containing 50 mM potassium phosphate, pH 7.2, 0.2 mM NADH, 10 μg purified OsMDHAR and 1 unit AsA oxidase (Sigma-Aldrich, USA), with increasing concentration of AsA and ISD. The Michaelis constant (Km) and maximum velocity (Vmax) were determined by fitting the Michaelis-Menten equation to the experimental data. Data represent averages of three independent measurements. The table lists the values of Km and Vmax.


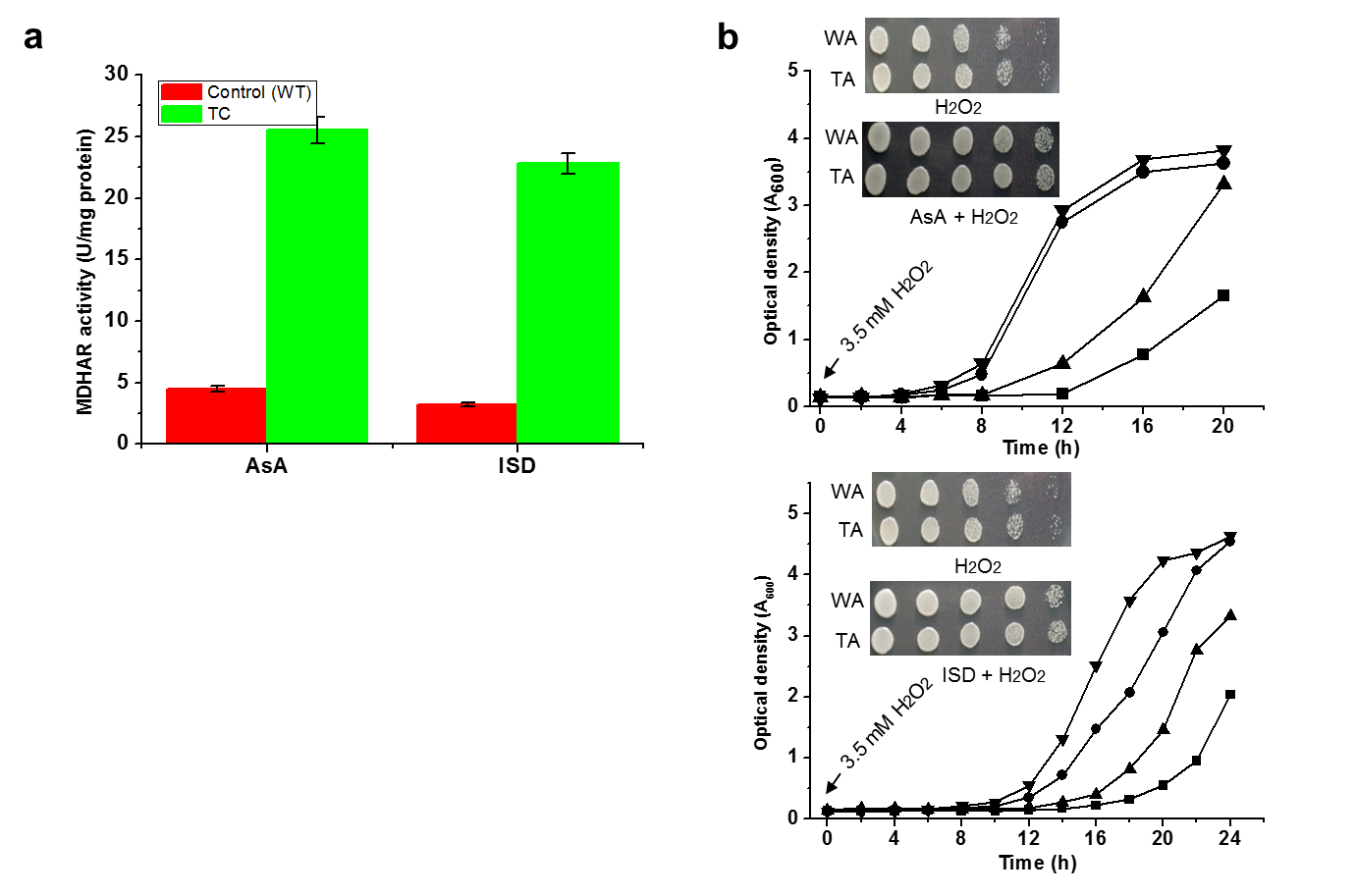


Figure S6. Substrate specificity and exogenous effect of ascorbate and its analogue under condition of oxidative stress. (a) Comparison of MDHAR enzyme activity from yeast crude extract using ascorbic acid (AsA) and isoascorbic acid (ISD). Control, yeast cells transformed an empty vector; TC, *OsMDHAR*-expressing yeast cells. (b) Stress response of *ara2△* yeast cells to oxidative stress in the presence of AsA (upper panel) and ISD (lower panel). For growth kinetics, precultured yeast cells were inoculated in YPD medium containing 3.5 mM H_2_O_2_ plus 2 mM AsA or 2 mM ISD, and optical density measured at 600 nm at 2-h intervals for the indicated time. Cell viability was also monitored by a spotting assay. Log-phase yeast cells (A_600_ ≈ 1.5) were treated with 10 mM H_2_O_2_ for 1 h at 28 ºC with shaking, and serially diluted with YPD medium. Five microliters were spotted onto YPD agar plates, which were then incubated for 3 days at 28 °C, and photographed. WA, *ara2△* yeast cells with an empty vector; TA, *ara2△* yeast cells expressing *OsMDHAR*;, TA cells with AsA or ISD; circle, WA cells with AsA or ISD; upward triangle, TA cells without AsA or ISD; square, WA cells without AsA or ISD; downward triangle. The results from the *ara2△* yeast cells show that the *ARA2* gene encoding D-erythroascorbic acid biosynthesis is deleted.

**Methods**

**UV-visible spectrum**

The UV-visible spectrum was recorded using a Multiskan GO microplate spectrophotometer (Thermo Fisher Scientific, USA). The experiments were carried out in a reaction volume of 100 μL in 96-well plates at 25 °C. Protein concentration was 10 mg mL^-1^ and 5 mM NADH was added to the solution.

**Isothermal titration calorimetry (ITC) experiments**

ITC experiments were conducted using an Auto-ITC200 (GE healthcare, USA) with a cell volume of 370 μL. Cell samples contained 0.07–0.14 mM OsMDHAR variants in 20 mM Tris-HCl, pH 8.0, 200 mM NaCl and were titrated using 0.7–1.0 mM NAD(P)H by performing 20 or 40 injections. After subtraction of the baseline, the integrated heat responses were fitted to the single binding site model using the ORIGIN software package supplied with the calorimeter.

**MDHAR activity**

MDHAR activity was assayed spectrophotometrically. The assay was performed at 25 °C with a reaction mixture (1 mL) containing 50 mM potassium phosphate, pH 7.2, 0.2 mM NADH, 2 mM AsA or 2 mM ISD, 1 unit AsA oxidase (Sigma-Aldrich, USA), and crude extract. The absorbance was measured at 340 nm by monitoring NADH oxidation, and the activity was calculated using an absorbance coefficient of 6.2 mM^−1^cm^−1^. One unit is the amount of enzyme that oxidizes 1 nmol of NADH per min at 25 °C. Enzyme activity was represented relative to WT proteins, which was defined as 100 % [^1^](#_ENREF_1).

**Exogenous effect of ascorbate and its analogue on oxidative stress**

Yeast cells pre-cultured overnight were inoculated (1 % of culture volumes) in fresh YPD medium containing 1 % yeast extract, 2 % peptone, and 2 % dextrose, and used for subsequent experiments. For growth kinetics, yeast cells (*ara2△*) were cultured in YPD medium containing 3.5 mM H_2_O_2_ in the absence and presence of 2 mM ascorbic acid (AsA) and 2 mM isoascorbic acid (ISD) and monitored by measuring optical density at 600 nm at 2-h intervals for the indicated time. For the spotting assay, mid-log phase yeast cells (A_600_ ≈ 2.0) were pretreated with 10 mM AsA and 10 mM ISD for 1 h at 28 °C with shaking, washed twice with YPD medium to remove residual AsA and ISD, treated with 10 mM H_2_O_2_ for 1 h at 28 °C with shaking, and then serially diluted with YPD medium. Five microliters of the diluted solutions were spotted onto YPD agar plates.

**References**

1 Eltayeb, A. *E. et a*l. Overexpression of monodehydroascorbate reductase in transgenic tobacco confers enhanced tolerance to ozone, salt and polyethylene glycol stresses*. Plan*t**a 2**25, 1255-1264, doi:10.1007/s00425-006-0417-7 (2007).
